# Pubertal development of the understanding of social emotions: Implications for education

**DOI:** 10.1016/j.lindif.2010.05.007

**Published:** 2011-12

**Authors:** Stephanie Burnett, Stephanie Thompson, Geoffrey Bird, Sarah-Jayne Blakemore

**Affiliations:** a UCL Institute of Cognitive Neuroscience, London, UK; b School of Psychology, Birkbeck College, London, UK

**Keywords:** Puberty, Emotion, Adolescent brain development, Educational neuroscience, Self-awareness

## Abstract

Recent developmental cognitive neuroscience research has supported the notion that puberty and adolescence are periods of profound socio-emotional development. The current study was designed to investigate whether the onset of puberty marks an increase in the awareness of complex, or “mixed,” emotions. Eighty-three female participants (aged 9–16 years) were divided into three groups according to a self-report measure of puberty stage (early-, mid- and post-puberty). Participants were presented with emotional scenarios, and used four linear scales to rate their emotional response to each scenario. Scenarios were designed to evoke social emotions (embarrassment or guilt) or basic emotions (anger or fear), where social emotions are defined as those which require the representation of others' mental states. We measured the relative complexity or “mixedness” of emotional responses, that is, the degree to which participants reported feeling more than one emotion for a given scenario. We found that mixed emotion reporting increased between early- and post-puberty for social emotion scenarios, and showed no relationship with age, whereas there was no change in mixed emotion reporting for basic emotion scenarios across age or puberty groups. This suggests that the awareness of mixed emotions develops during the course of puberty, and that this development is specific to social emotions. Results are discussed in the context of brain development across puberty and adolescence, with speculation regarding the potential implications for education.

## 1 Introduction

“Neuroscience has the potential to make important contributions to education. These potential contributions are of at least three kinds: novel understanding about the biological and environmental processes determining learning; the identification of neural markers for educational risk; and neural methods for evaluating different teaching approaches, remediation packages or educational debates” (UK Government *Foresight* Project Report SR-E1, p2).

Knowledge of how the brain learns could, and will, have a great impact on education. Understanding the brain mechanisms that underlie learning and memory, and the effects of genetics, the environment, emotion and age on learning could transform educational strategies and enable us to design programmes that optimize learning for people of all ages and of all needs. Only by understanding how the brain acquires and lays down information and skills will we be able to reach the limits of its capacity to learn. Neuroscience can now offer some understanding of how the brain learns new information and processes this information throughout life (see Blakemore & Frith, 2005).

Understanding the neural basis of socio-emotional functioning, and how social and emotional factors interact with the capacity to learn, is also a crucial concern to education. Social functioning plays a role in shaping learning and academic performance (as well as vice versa), and thus understanding the neural basis of social behaviour may contribute to understanding the origins of educational success and failure. It can also facilitate an understanding of how children with additional socio-emotional needs can be included in mainstream schools, and how to reduce exclusion. In this paper, we describe an example of an area within cognitive neuroscience that might one day have profound implications for education, that is, socio-emotional development during puberty and adolescence. In this example we focus on an empirical study in which the aim was to investigate how the understanding of emotional complexity, or “mixedness”, develops across puberty, for two distinct categories of emotion: social and basic emotion.

### 1.1 Social emotion and mixedness

Social emotions, such as embarrassment, guilt and shame, can be defined as emotions that require the representation of another social agent's mental states (*e.g.* their opinion or feelings; Burnett, Bird, Moll, Frith, & Blakemore, 2009, see also Olsson & Ochsner, 2008). In order to feel embarrassed, for example, you must believe that another social agent thinks your actions are foolish. In contrast, basic emotions, such as fear and disgust, minimally constitute immediate affective reactions that do not require consideration of the mental states of others. Studies have indicated that the understanding of basic emotions emerges earlier in development than the understanding of social emotions. For example, Harris, Olthof, Terwogt, and Hardman (1987) showed that a sample of five year old children were able to think of situations in which a basic emotion would be felt, but not situations in which a social emotion would be felt. By the age of seven, children were able to think of situations that would plausibly elicit social emotions, such as pride, jealousy and guilt.

The development of social emotion understanding beyond middle childhood is not well characterised. However, the period from middle childhood to adolescence is thought to be accompanied by an increase in multi-dimensional or abstract thinking about other people, and this includes thinking about the diverse factors that influence people's thoughts and feelings (Inhelder & Piaget, 1958; Wainryb, Shaw, & Maianu, 1998). Therefore, this age period is likely to be accompanied by an increasingly complex understanding of emotions in self and in others. Furthermore, evidence from behavioural and neuroimaging studies suggest that the interpersonal, physical and hormonal changes associated with puberty may contribute to changes in the behavioural and neural correlates of social emotion processing. This evidence will now be summarised.

### 1.2 Development of social emotion

Adolescence can be defined as the period between puberty onset and the attainment of a stable adult role (Lerner & Steinberg, 2004), and it represents a period of acute socio-emotional change (although see Choudhury, 2010). Self-awareness and the self-concept undergo profound development during the adolescent years (Sebastian, Burnett, & Blakemore, 2008), and the perceived opinions of peers are particularly important during this time in shaping the self-concept and in modulating social behaviour (Brown, 2004). There is evidence that social and emotional development may be linked to pubertal development as well as age *per se,* as self-report studies show that young adolescents (particularly girls), from early to mid-puberty are more self-conscious than both pre-pubescent children and post-pubescent adolescents (Elkind & Bowen, 1979; Rosenberg & Simmons, 1975; Simmons, Rosenberg, & Rosenberg, 1973). It has been suggested that the physical changes of puberty contribute to developmentally enhanced self-consciousness, through a mechanism of heightened vulnerability to environmental circumstances that threaten the self-image (Simmons et al., 1973). Thus, mid-puberty may be a time of particular sensitivity to social or self-conscious emotions, such as embarrassment and shame.

### 1.3 Structural brain development across adolescence and puberty

Recent brain imaging studies are not inconsistent with the notion that social emotion processing and/or mixed emotion understanding may continue to develop during puberty and adolescence. Structural neuroimaging studies in humans have demonstrated that prefrontal and temporal brain regions supporting emotion understanding and self-awareness undergo protracted anatomical development, until late adolescence and beyond (Giedd et al., 1999; Gogtay et al., 2004; Shaw et al., 2008; Sowell et al., 1999). In particular, there seems to be a reorganisation of cortical grey matter that coincides with the onset of puberty (Giedd et al., 1999; Shaw et al., 2008; see Blakemore, Burnett & Dahl, 2010). Specifically, in the frontal lobe, grey matter density increases during childhood, reaching its peak at around puberty onset (approximately 11 years in girls and 12 years in boys), and this is followed by a protracted reduction in grey matter during adolescence. Limbic brain regions such as the amygdala and hippocampus show protracted increases in volume throughout adolescence (Giedd et al., 1997; Ostby et al., 2009). In contrast, in basic sensory processing regions of the brain peak grey matter volume is attained prior to adolescence (Giedd et al., 1999; Giedd, 2004; Sowell et al., 1999; Paus, 2005, 2008).

The majority of these MRI studies include no measure of puberty stage. However, studies in non-human animals suggest that the onset of puberty marks a crucial phase of structural reorganisation in the brain, affecting social and motivational behaviours (Cahill, 2006; Romeo, 2003). In addition, recently, a small number of cross-sectional anatomical MRI studies have been published that include measures of puberty (for a review, see Blakemore, Burnett, & Dahl, 2010). For example, Neufang et al. (2009) showed that grey matter volume in the amygdala varied as a function of circulating testosterone and oestrogen, as well as Tanner Stage of puberty (Tanner, 1971). Perrin et al. (2009) showed evidence that self-reported puberty stage correlates with white matter volume in males. This suggests that puberty stage, as well as chronological age *per se*, is an important factor in the structural development of the brain during adolescence, including within brain regions implicated in emotion, social processing and self-awareness.

### 1.4 Functional brain development

**Fig. 1:**
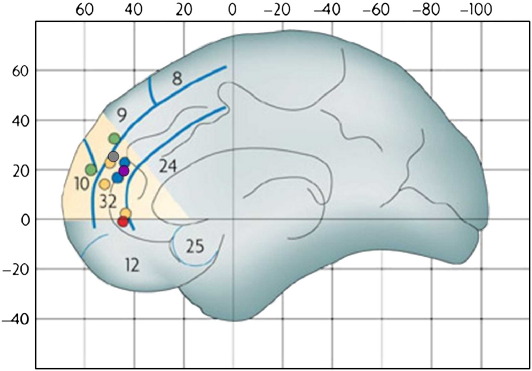
Several recent studies have shown that activity in DMPFC (pale yellow area) during social processing decreases between adolescence and adulthood. The purple dot shows a region activated more during social than basic emotion processing in adolescents than in adults (Burnett et al., 2009); green: activity greater during sarcasm comprehension in adolescents (Wang, Lee, Sigman, & Dapretto, 2006); blue: activity greater during mental state self-insight in adolescents (Blakemore et al. 2007); yellow: activity greater during self-other evaluation (Pfeifer et al., 2007); red: increase in activation with age during adolescence while watching social cartoons (Moriguchi et al., 2007).

In addition to changes in the structure of the brain, puberty and adolescence is accompanied by changes in functional measures within brain regions involved in emotion and social awareness. For example, functional MRI studies have shown changes in activity within the mentalising system across adolescence (Blakemore, 2008; Pfeifer, 2009). Specifically, adolescents show greater activity than do adults within anterior rostral medial prefrontal cortex (arMPFC), a brain region involved in representing mental states (including feelings, beliefs and desires), during social cognition relative to control tasks (see Fig. 1 for meta-analysis); including social cognition tasks explicitly assessing self-other-awareness (*e.g.* how others see you; Pfeifer et al., 2009). In one fMRI study conducted in our laboratory, adult (age 22–32) and adolescent (age 10–18) participants read scenarios that described either social or basic emotions. In both age groups, thinking about social emotions relative to basic emotions activated regions within the “social brain network” (Frith & Frith, 2003), including dorsal medial prefrontal cortex (DMPFC), superior temporal sulcus (STS) at the temporo-parietal junction (TPJ) and the temporal poles. However, there were striking differences between the groups. Activity in DMPFC, a brain region involved in representing mental states, was higher during social emotion relative to basic emotion in the adolescent group than in the adult group. Activity in the left temporal pole, a brain region involved in representing semantic social–emotional information (*e.g.* Olson et al., 2007), showed the reverse developmental pattern, being more highly activated in adults than in adolescents for social emotions. This finding indicates that the neurocognitive strategy for understanding social emotions develops with age, with relative activity moving from frontal regions to temporal regions between adolescence and adulthood.

A number of studies show evidence for a relationship between puberty and functional brain activity during social–emotional tasks. For example, fMRI studies comparing typical children and adolescents to those with endocrine disorders (*e.g.* precocious puberty) indicate a relationship between adrenal hormone levels and amygdala activity during emotional face processing in females (Ernst et al., 2007), and between testosterone levels and hippocampal responsiveness during emotional face processing in males (Mueller et al., 2009). One recent study in hormonally typical adolescent females has demonstrated a link between puberty stage and functional activity within the ventrolateral prefrontal cortex and amygdala during an emotional face processing task (Forbes, Phillips, Ryan, Dahl, 2011). Thus, evidence from fMRI studies lends support to the notion that there is a relationship between puberty hormones and the cognitive and neural processing of social–emotional information, via effects on the brain.

In summary, there is evidence that puberty stage influences social–emotional processing via psychological effects on the self-image, changes in social–emotional awareness, and effects on the structure and function of the brain. However, pubertal changes in social–emotional awareness are not well characterised. Therefore, the current study investigated the development of social emotion understanding across puberty. Specifically, and in order to limit the scope of this wide subject area, the current study focussed on a single aspect of emotion understanding, the understanding of mixed emotion.

### 1.5 Development of mixed emotion

Mixed emotion understanding is the ability to acknowledge that a number of discrete emotions may be simultaneously elicited by a single event. Mixed emotions can be similar in valence, such as feeling both anger and disappointment at a missed opportunity. Mixed emotions can also be oppositely valenced. For example, one could feel both anger and relief upon the safe return of a wayward child. There is a body of research on the understanding of mixed emotions in early childhood (Harris, 1989; Harter & Buddin, 1987; Larsen, To, & Fireman, 2007). Studies using structured interviews have shown that children around the age of five deny that mixed emotions occur, or are even possible (Harter, 1977, 1983; Harter & Buddin, 1987; Harris, 1983; Larsen, To, & Fireman, 2007). For example, Harter and Buddin (1987) asked children between the ages of three and 11 to describe situations that would make them feel either opposite valence (happy, sad) or same valence (happy, excited) emotions at the same time. Children aged five reported statements such as “You'd have to be two different people to have two feelings at the same time”. Between the ages of six and eight, children initially succeeded in describing situations in which two emotions would be felt in rapid succession, and subsequently, were able to describe situations in which two same-valence emotions would be experienced at the same time. Only between the ages of 10 and 11 were children able to describe situations in which two opposite valence emotions would be felt simultaneously.

A recent study by Larsen et al. (2007) investigated mixed emotion in children aged five to 12 years. Children were presented with a video clip from a children's fairy tale (The Little Mermaid), which culminated in a “bittersweet” scene in which a father character is portrayed as having to say farewell to his daughter. Children were asked: (a) how the father character would feel, and (b) how the clip made them feel. Children were given structured prompts to generate more than one emotion, and if they were able to do this, were then asked to indicate whether the emotions would be experienced sequentially or simultaneously (mixed emotions). Results showed a significant linear effect of age on measures of both the tendency to report that the father would experience mixed emotions, and the tendency to report personally experiencing mixed emotions. These data indicate that, in addition to developing a better conceptual understanding of mixed emotion in others (the father), older children also demonstrate a greater tendency to report mixed emotions in themselves.

The development of mixed emotion understanding during puberty has not previously been studied. However, as described above, puberty and adolescence is a period of social–emotional change and emerging self-consciousness and self-awareness. This may lead to development in mixed emotion understanding across puberty, particularly for the understanding of social or self-conscious emotions.

It should be noted here that the conceptual understanding of one's own mixed emotions may be distinct from actually experiencing mixed emotions. Emotional ambivalence, that is, the expression of both positive and negative emotions simultaneously is shown by infants aged around one year (Ainsworth, Blehar, Waters, & Wall, 1978; see also Campos, Barrett, Lamb, Goldsmith, & Stenberg, 1983). The observation of a developmental time lag between displaying versus reporting mixed emotions has prompted the suggestion that mixed emotion understanding emerges as children learn to interpret the emotional reactions that they already express, through an increasingly mature appreciation of the complex causal relationships between situations and emotions (Harris, 1989). In the current study, we were primarily concerned with the understanding of mixed emotions, insofar as they can be measured by self-report.

### 1.6 The current study: the understanding of mixed emotions during puberty

To our knowledge, no study to date has investigated the understanding of mixed emotion across puberty. As indicated above, there is evidence that pubertal development may influence emotional awareness via psychological mechanisms and via effects on the brain. Therefore, it is reasonable to hypothesise changes in mixed emotion understanding across puberty, especially for social emotions.

In the current study, we investigated the understanding of mixed emotions across puberty, for both social and basic emotions. We tested 83 participants between the age of 9.5 and 16.33 years, divided into three Puberty Groups (Early-, Mid- and Post-puberty). Given our wide age range, we expected chronological age to differ between puberty groups. During the experiment, participants at pre- mid and post-puberty stages were instructed to imagine a series of emotional scenarios, and to report how strongly they would feel each of four emotions (anger, fear, embarrassment and guilt) in response to each scenario. Ratings along the four emotion scales were used to develop a measure of mixedness in emotional responding. It was hypothesised that mixed emotion understanding would develop across puberty.

The scenarios were designed to evoke primarily social emotions (embarrassment and guilt) or primarily basic emotions (anger and fear). Given our definition of social emotion, social emotions can be conceptualised as inherently more mixed than basic emotions (social emotions require consideration of the mental states of more than one agent). Thus, we expected to see more highly mixed emotional responses for social relative to basic emotion scenarios overall. However, given the evidence cited above, we hypothesised that mixed emotional responses to social emotion scenarios in particular would continue to develop across puberty.

## 2 Methods

### 2.1 Participants

Eighty-three female participants aged 9.5 to 16.33 years took part in the study. Participants were recruited via parental letters distributed by two urban state schools, a secondary school and adjacent primary feeder school. Parental consent was obtained prior to testing. None of the participants had a known diagnosis of autism or dyslexia, or any other diagnosed developmental disorder. The study was approved by the local ethics committee.

We included female participants only, for the following reasons. Firstly, it is reported that there are differences between the sexes in expressing emotions (Kring & Gordon, 2007), which could impact on our self-report measure. Second, there are sex differences both in the structure of brain regions involved in emotion and social cognition (Lenroot et al., 2007; Schmithorst, Holland, & Dardzinski, 2008), and in their activation during social and emotional tasks (Hall, Witelson, Szechtman, & Nahmias, 2004; Killgore & Yurgelun-Todd, 2004; Moll, Zahn, de Oliveira-Souza, Krueger, & Grafman, 2005; Moll et al., 2005). Finally, the temporal profile of puberty, and its impact on social behaviour, differs between the sexes (Savin-Williams & Weisfeld, 1989, cited in Spear 2000; Giedd et al., 1999; Romeo, 2003).

Participants were divided into three Puberty Groups on the basis of a physical development questionnaire adapted from Carskadon and Acebo (Carskadon & Acebo, 1993; Tanner, 1971; Tanner & Whitehouse, 1976): Early-puberty (*N* = 23, mean (s.d.) age = 11.7 (1.44), range = 9.5–14.08), Mid-puberty (*N* = 40, mean (s.d.) age = 13.0 (1.15), range = 10.5–15.33) and Post-puberty (*N* = 20, mean (s.d.) age = 15.2 (0.70), range = 13.67–16.33). Chronological age differed across Puberty Groups (one-way ANOVA: *F*_2,82_ = 49.914, *p* < 0.001).

The physical development questionnaire was completed by secondary school age participants in a quiet room in school, and was posted to parents of primary school age participants for completion by the parent alone, the parent and child together, or the child alone, according to parental preference. The questionnaire comprised self-report items assessing age, height, weight, menarche (yes/no), three secondary sexual characteristics: growth spurt, bodily hair and skin changes (each had four response options: not yet started, just started, definitely started, finished), and stage of development relative to same-age peers (five response options). We assigned participants to the Early-puberty group if they circled “no” in response to the question “Have you started having periods?” and in response to the questions regarding secondary sexual characteristics (growth spurt, skin changes and underarm hair). We assigned participants to the Post-puberty group if they circled “yes” in response to the question “Have you started having periods?” and in response to questions regarding secondary sexual characteristics (growth spurt, skin changes and underarm hair). Participants who did not fit either of these criteria were assigned to the Mid-puberty group.

### 2.2 Procedure

**Fig. 2:**
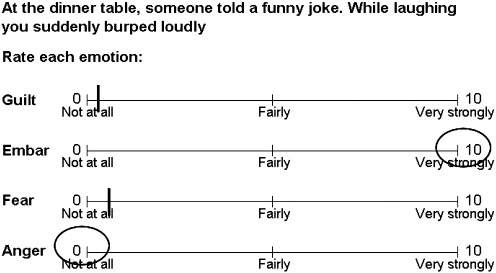
Example of a task scenario. Each was read aloud to participants, who were asked to imagine how they would feel if the scenario happened to them, and then to rate how strongly they would feel each of the four emotions, on a scale of 0 (not at all) to 10 (very strongly).

Participants were tested in groups of two to four in a quiet room in school, seated so they were unable to see each others' papers or confer. Participants were each given a paper-based task containing a series of sentences with attached emotion rating scales (see Fig. 2). The experimenter instructed participants that they would be reading a number of sentences describing situations in which they might feel emotions such as anger, fear, embarrassment and guilt. Before commencing the experiment, the experimenter defined each of the four emotions, giving examples of situations in which each might be felt (see Table 1, second column).

During the experiment, participants silently read the scenarios and, at the same time, the experimenter read each scenario aloud to participants. Participants were instructed to imagine how they would feel in each scenario, and to rate their emotional response using the four rating scales for anger, fear, embarrassment and guilt. Participants were told that they could place a mark anywhere they wished on the analogue scale, either by circling 0 (not at all) or 10 (very strongly), or by placing a mark anywhere in between 0 and 10 (see Fig. 2).

Each scenario was designed to evoke primarily one of four emotions: anger, embarrassment, fear or guilt. However, we designed the stimuli such that some sentences might reasonably evoke more than one emotion. The task consisted of 32 scenarios in total, of which 16 were primarily Basic emotion scenarios (eight anger, eight fear) and 16 were primarily Social emotion scenarios (eight embarrassment, eight guilt). Examples of emotion scenarios are given in Table 1 (third column). The order of emotion words in the rating section for each scenario was counterbalanced between participants. The order of emotions in the instruction phase and the order of scenarios in the task were counterbalanced between the small groups of simultaneously-tested participants.

### 2.3 Analysis

Each scenario was rated along four 10 cm emotion scales for anger, fear, embarrassment and guilt. In order to investigate mixed emotional responses, we developed a measure that encapsulated the extent to which participants used all four rating scales for Social and Basic emotion scenarios. We measured the distance of the emotion ratings along each of the four 10 cm scales for each scenario, which were therefore non-integer values ranging from 0 to 10 cm. Next, we coded each scenario according to the number of times a rating > 2 cm was given. This was an ultimately arbitrary cut-off point, but was chosen on the basis that ratings below 2 cm indicate very weak reported feelings of a particular emotion. Rating response codes were as follows:

Rating code 0: none of the four emotions rated > 2 cm
Rating code 1: one of the four emotions rated > 2 cm
Rating code 2: two of the four emotions rated > 2 cm
Rating code 3: three of the four emotions rated > 2 cm
Rating code 4: four out of four emotions rated > 2 cm

**Fig. 3:**
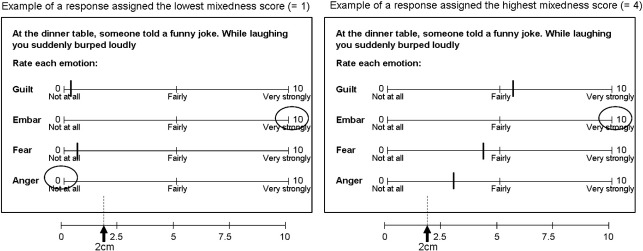
Scoring of responses. Each response (32 per participant: 16 Social, 16 Basic) was given a mixedness score that took into account how many times a rating > 2 cm had been given. A maximally mixed emotional response (above, right) was one in which all four emotions were rated > 2 cm. We compared mean mixedness scores for Social and Basic emotion scenarios across Puberty Groups.

A response assigned a rating code of 4 is maximally mixed, since all four emotions are rated > 2 cm (see Fig. 3). A rating code of 1 indicates a minimally mixed response, since only one of the four emotions is rated > 2 cm. Responses assigned a rating code of zero, *i.e.* in which no emotion was rated > 2 cm, were omitted from the analysis, since it was considered that this response pattern indicated a lack of emotion.

For each participant, we calculated the mean mixedness score for Social and Basic emotion scenarios separately, resulting in a range of possible mean mixedness scores between 4 (maximum mixedness) and 1 (minimum mixedness). After checking for normality and excluding outliers lying > 3 s.d. from the group mean for Social and Basic scenarios separately (one Basic, Post-puberty mixedness score excluded), we conducted mixed design repeated measures 3 × 2 ANOVA to test for group differences in mean mixedness scores for Social and Basic emotion scenarios. There was one between-subjects factor with three levels: Puberty Group (Early-puberty, Mid-puberty and Post-puberty); and one within-subjects factor with two levels: Emotion Type (Social, Basic). Because chronological age differed across Puberty Groups, we explored possible effects of age on mixedness scores by conducting linear regression between age and Social and Basic mixedness scores. We applied a threshold for significance at *p* < 0.05 throughout, and used *post hoc* Bonferroni-corrected *t*-tests to explore paired differences.

## 3 Results

**Fig. 4:**
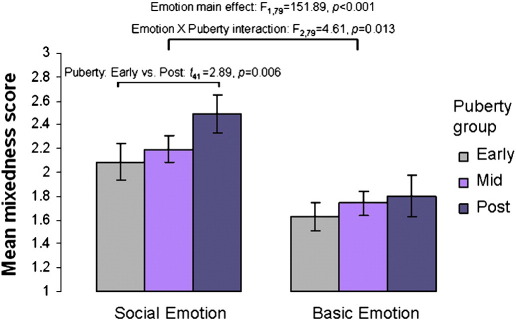
Mean mixed emotion score for Social (left) and Basic (right) emotion scenarios in each Puberty Group. The maximum possible mixedness score was 4 (four emotions rated > 2 cm) and the minimum was 1 (one emotion rated > 2 cm). There was an interaction between Puberty Group and Emotion driven by higher mixedness scores for Social relative to Basic emotions in the Post-puberty group relative to the Early-puberty group.

Mean mixedness scores for Social and Basic emotion scenarios by Puberty Group are shown in Fig. 4. Skew and kurtosis for Social and Basic mixedness scores were between + 2 and −2, indicating that the data were distributed satisfactorily close to normal. We excluded one Post-puberty outlier > 3 s.d. from the group mean for Basic mixedness. Repeated measures ANOVA revealed a main effect of Emotion Type on mixedness scores (*F*_1,79_ = 151.89, *p* < 0.001) such that more highly mixed emotional responses were given for Social than for Basic emotion scenarios (Social: mean (s.d.) mixedness = 2.21 (0.70); Basic: mean (s.d.) mixedness = 1.68 (0.61)). Paired samples *t-*tests showed that mixedness for Social Emotion scenarios was significantly higher than mixedness for Basic Emotion scenarios in all three Puberty Groups (Early-puberty: *t*_22_ = 6.82, *p* < 0.001; Mid-puberty: *t*_39_ = 7.71, *p* < 0.001; Post-puberty: *t*_19_ = 7.02, *p* < 0.001).

There was no main effect of Puberty Group (*F*_2,79_ = 1.901, *p* = 0.16). However, there was a significant interaction between Puberty Group and Emotion Type (*F*_2,79_ = 4.614, *p* = 0.013). The interaction was driven by higher mixedness scores in the Post-puberty group than in the Early-puberty group for Social Emotion scenarios (*t*_41_ *=* 2.89, *p* = 0.006), but not for Basic Emotion scenarios (*t*_40_ = 1.07, *p =* 0.291). No other comparisons reached significance. There was no relationship between chronological age and mixedness scores for Social or Basic Emotion scenarios (linear regression between age and Social Emotion mixedness: *p* = 0.205; age and Basic Emotion mixedness: *p* = 0.796).

## 4 Discussion

Previous research has demonstrated that the ability to acknowledge that a number of discrete emotions may be elicited by a single event, or an awareness of “mixed” emotion, first emerges in middle childhood and continues to develop up to age 12 (Harris, Donnelly, Guz, & Pitt-Watson, 1986; Harter, 1983; Harter & Buddin, 1987; Larsen et al., 2007). On the basis that pubertal development is related to social awareness and self-conscious affect (Rosenberg & Simmons, 1975; Simmons et al., 1973), and in view of evidence that puberty hormones organise the structure and function of the brain, including regions of the brain involved in social behaviour and emotion (Cahill, 2006; Neufang et al., 2009; Peper et al., 2009, 2010; Romeo, 2003; Schulz, Molenda-Figueira, & Sisk, 2009), the current study investigated mixed emotion understanding across puberty. We assessed mixed emotion understanding by taking a measure of the number of discrete emotions reported in response to imagined social or basic emotion scenarios.

### 4.1 Mixedness in social emotion

Our results revealed two main findings. First, and as predicted, responses were significantly more mixed for social emotions than for basic emotions, across puberty groups. We define social emotions, for example guilt and embarrassment, as emotions that require the representation of another person's mental states (Burnett et al., 2009; Olsson & Ochsner, 2008). In contrast, no mental state representation is required to experience basic emotions. By this definition, social emotions are more representationally complex, and more inherently “mixed” than basic emotions, since social emotions require consideration of multiple mental perspectives. Furthermore, emotions such as guilt and embarrassment involve the consideration of social norms and the implications of one's actions on others. For example, a guilt-inducing situation is one that involves thinking about one's own feelings, intentions and actions (resulting perhaps in anger and shame directed at the self), as well as the feelings of the wronged party, and perhaps the real or imagined opinions of a judging third party. In contrast, the feeling of fear minimally involves an immediate emotional reaction to a threatening stimulus. Therefore, we consider that the greater mixedness in emotional responding to social emotion scenarios than to basic emotion scenarios across puberty groups reflects the greater inherent emotional complexity of social relative to basic emotions, although this conclusion warrants further theoretical and empirical investigation. For example, studies should directly assess the developmental relationship between mentalising ability (*e.g.* Dumontheil, Hassan, Gilbert, Blakemore, in press), and the understanding of mixed emotional responses in social emotion situations.

### 4.2 Development of mixed emotion understanding

The second result in the current study was the interaction between puberty group and emotion condition. We found that the degree of mixedness in the emotional response to social, but not basic, emotion scenarios increased between early- and post-puberty. This novel result provides evidence for the continued development of an understanding of mixed emotion across the period of puberty. Our result suggests that this development is specific to social emotions. This finding is in line with, and an extension of previous research showing that the understanding of basic emotions precedes the understanding of social emotions developmentally, and that mixed emotion understanding continues to develop during childhood and up to the age of 12 (Harris et al., 1986; Harter & Buddin, 1987; Larsen, To, & Fireman, 2007). Our results show no change in mixed emotion reporting for basic emotions in females after late childhood/early-puberty (mean age 11 years in the current sample), but a change in mixed emotion reporting for social emotions into late puberty, and up to the age of 16.

### 4.3 Caveats and limitations

There are a number of caveats and limitations in the current study. One caveat is the restriction of our sample to females: Mixed emotion should also be investigated across puberty in males to complete the developmental picture.

Another notable caveat is that, although there was no evidence for a relationship between age and mixed emotional responses, the relationship between age and puberty group within our sample limits the interpretation of our findings with regards the effects of puberty specifically. Puberty stage is strongly related to chronological age between 9 and 16 years in typical female samples (Rubin et al., 2009). Therefore, this issue should be addressed by conducting a study across a more restricted age range, for example in a group of females aged 12 at pre- mid- and post-puberty stages (Rubin et al., 2009).

Another caveat in the current study is with our method of assessing emotional mixedness, which requires validation. Previous studies of mixed emotion understanding have used structured interview techniques to show results that are consistent with the current finding (Harris, 1989; Harter, 1983; Harter & Buddin, 1987; Larsen et al., 2007). Another study has shown age differences across adulthood in mixed emotion, specifically, in self-reported discomfort in response to watching adverts that elicit mixed emotions between young adulthood (mean age 22 years) and older adulthood (mean age 73 years) (Williams & Aaker, 2002). The current finding would benefit from replication in 9–16 year old females using structured interviews, and our self-report measure of mixedness should be validated in other age groups.

A limitation of the current study, and one that applies in general to studies that investigate relationships between some dependent measure and puberty stage, relates to the conceptual and methodological issues with measuring puberty. These issues have been addressed in detail elsewhere (Blakemore, Burnett, & Dahl, 2010; Dorn et al., 2006). Briefly, puberty “is not an event or unitary process” (Dorn et al., 2006), but comprises several distinct processes that extend across several years culminating in reproductive maturity. These processes include activation of adrenal, gonadal, and growth hormone systems, but in addition a variety of direct and indirect effects, from growth spurt, to changing self-image. The current study used a parental/self-report puberty measure that collapsed across adrenal (*e.g.* bodily hair), gonadal (menarche) and growth hormone (growth spurt) items. Other studies have used clinician-assessed Tanner stage, hormonal assays, or a combination of techniques (Dorn et al., 2006; Shirtcliff, Dahl & Pollak, 2009; Blakemore, Burnett, and Dahl, 2010). At present there is no consensus on what is the “gold standard” for assessing puberty stage; it has been argued that this is dependent on the particular research question (see *e.g.* Dorn et al., 2006).

### 4.4 Interpretation of development in mixed emotion understanding

The development across puberty in mixed emotion responding for social emotion scenarios could be due to a number of factors, as discussed in the introduction. One factor, which has received increased attention in recent years, is the neuroanatomical reorganisation which occurs during puberty and adolescence. A number of studies have shown that prefrontal and temporal brain regions supporting emotion and mental state understanding, and self-awareness, show protracted neuroanatomical development, with a reorganisation of grey matter during at around puberty (Giedd et al., 1999; Gogtay et al., 2004; Shaw et al., 2008; Sowell et al., 1999). In addition, a number of recent studies have shown relationships between puberty variables (*e.g.* hormone concentrations and Tanner stage) and structural brain measures (*e.g.* Neufang et al., 2009). Changes in neural structure, as shown in MRI studies, are thought to result in changes in the information processing carried out in these regions, and in the resultant cognitive and behavioural capabilities of the developing individual (Johnson, Grossman, & Cohen Kadosh, 2009; Olesen, Nagy, Westerberg, & Klingberg, 2003; Olson et al., 2009; Schmithorst, Wilke, Dardzinski, & Holland, 2005; Stevens, Kiehl, Pearlson, & Calhoun, 2007, 2009). This may correspond to results from functional neuroimaging studies, which have shown a change across adolescence in patterns of functional activation during social emotion tasks (*e.g.* Burnett & Blakemore, 2009a; Burnett et al., 2009). In particular, fMRI studies have shown that activity in DMPFC decreases between adolescence and adulthood during social cognition (including social emotion) tasks, whereas activity in more posterior social/emotion-related regions shows the opposite developmental pattern (see Blakemore, 2008, for review; and Fig. 1 for meta-analysis). An implication of these findings could be that the period of puberty and adolescence, when DMPFC and other social brain regions are still developing functionally and structurally, might be a period of enhanced social malleability (Burnett & Blakemore, 2009b). An important question that remains is how neuroanatomical, behavioural and neurocognitive development across puberty and adolescence interact with environmental factors, such as the transition from middle to high school, and the influence of peer group norms and expectations.

### 4.5 Implications for education

This study presents preliminary evidence that the understanding of complexity, or “mixedness” in social emotions develops across puberty in females. Research is required to extend this finding in males, to evaluate our methodology, and to disentangle possible causes driving such socio-emotional development across puberty. Speculatively, the current findings, alongside other recent results in developmental cognitive neuroscience research, might have certain potential implications for education across the period of puberty and adolescence. One potential implication is that the increase in awareness of the inherent emotional complexity of social situations may represent a challenge on the attentional and processing resources of adolescents in the classroom. A study by McGivern, Andersen, Byrd, Mutter, and Reilly (2002) showed evidence for a pubertal “dip” in the ability to match words to emotional faces. This result was interpreted as showing that, at puberty, emotional information may cause a greater degree of interference with ongoing tasks than at earlier or developmental later stages. As discussed above, the pattern of greater relative neural activity within prefrontal social brain regions during social emotion processing in adolescence suggests that additional metabolic, and possibly cognitive, resources are needed to complete such tasks. Therefore, it is possible that highly emotionally charged social situations in the educational environment could interfere with the mental resources that are devoted to learning.

Another implication of the current finding is that the development of the understanding of emotional mixedness in social situations may impact on or relate to changes in interpersonal sensitivity. Studies have shown that, during adolescence, relationships with peers become increasingly important and valued, as well as more complex and multi-layered (Brown, 2004; Csikszentmihalyi, Larson, & Prescot, 1977). In addition, there are changes in the understanding of the interpersonal consequences of expressing certain emotions to peers (Zeman, Cassano, Perry-Parrish, & Stegall, 2006), indicating a shift in the awareness of social situations and the feelings of others. Finally, the emotional impact of social inclusion and exclusion is heightened in early adolescence relative to older adolescence and adulthood (Sebastian, Viding, Williams, & Blakemore, 2010). This evidence indicates that puberty and adolescence is an important time in the development of emotional awareness, especially in the context of peer relationships. Therefore, early adolescence in particular could be a fruitful time for educational programmes to focus on empathy and peer support, for example through anti-bullying and extra-curricular policies.

The finding that changes in brain structure continue throughout puberty and adolescence has given rise to a recent spate of investigations into the way cognition (including social cognition) might change as a consequence. If early childhood is seen as a major opportunity–or a “sensitive period”–for teaching, perhaps so too should the teenaged years. During both periods, neural reorganisation is taking place. Perhaps the aims of education for adolescents might be adapted to include abilities reliant upon the parts of the brain that undergo the most dramatic changes during adolescence. These include executive function-related abilities such as internal control, multi-tasking and planning (Dumontheil Hassan, Gilbert, Blakemore, in press; Luna, Padmanabhan, & O'Hearn, 2010; Romine & Reynolds, 2005) — but also self-awareness (Sebastian et al., 2008), social cognitive skills such as the on-line use of mental state information (*e.g.* Dumontheil, Apperly, & Blakemore, 2009; Güroğlu, van den Bos, & Crone, 2009), and the understanding of complex social emotions, as shown in the current study. Finally, it could potentially be fruitful to include in the secondary education curriculum some teaching on the changes occurring in the brain during puberty and adolescence. Adolescents might be interested in, and could benefit from, learning about the changes that are going on in their own brains.

## Figures and Tables

**Table 1:** At the start of the session, the experimenter read aloud a definition and example of each emotion (column 2). During the emotion task, participants read a series of 32 emotion scenarios (examples in column 3) and rated their imagined emotional response using four rating scales.

Emotion	Definition and example	Example scenario from the task
Anger	When you feel mad and annoyed at someone or something, *e.g. You might feel annoyed if the bus is late and you know you are going to miss your appointment.*	You saw someone walk by the window. They threw rubbish into your garden.
Embarrassment	When you feel you look silly in front of people *e.g. You trip and fall in front of your friends.*	You ate too much cake at a party. You threw up in the living room in front of everyone.
Fear	When you feel scared *e.g. You feel scared when you are walking in a dark quiet street.*	You were riding your bike down a hill. Suddenly your brakes stopped working.
Guilt	When you feel that you have done something wrong and feel bad about it *e.g. You broke your friend's mobile phone.*	You were meant to look after your little brother but you went out. When you got back he was crying.
